# The validity of the diabetes self-management questionnaire (DSMQ) in Hungarian patients with type 2 diabetes

**DOI:** 10.1186/s12955-020-01595-7

**Published:** 2020-10-19

**Authors:** Agnes Vincze, Antonia Losonczi, Adrienne Stauder

**Affiliations:** 1 Department of Internal Medicine and Oncology, Semmelweis University, Korányi S. str. 2/a, Budapest, 1083 Hungary; 2 Faculty of Education and Psychology, Eötvös Lorand University, Budapest, Hungary; 3 Institute of Behavioural Sciences, Semmelweis University, Budapest, Hungary

**Keywords:** Type 2 diabetes mellitus, DSMQ, Self-management, HbA1c, Validity

## Abstract

**Background:**

A wide range of measuring instruments are available for diabetes self-management. According to several studies, a relatively new instrument, the diabetes self-management questionnaire (DSMQ), shows a consistent relationship with outcomes of diabetes treatment as well, such as glycated hemoglobin (HbA1c). Furthermore, the questionnaire is short, but covers the main aspects of diabetes management. Given the increasing prevalence of diabetes in Hungary, our goal was to adapt this user-friendly questionnaire and analyze its validity.

**Methods:**

After the standard translation process, we analyzed a sample of 221 people. The construct validity of the questionnaire was tested with HbA1c and body mass index. Morisky Medication Adherence Scale-8 values were tested via Pearson correlations. Known-groups validity of the DSMQ in relation to groups based on glycemic control levels was investigated using one-way ANOVA.

**Results:**

The “sum scale” of the questionnaire and the HbA1c values show an inverse relationship (r = − 0.253, *p* < 0.01). Body mass index was related to the “sum scale” (r = − 0.214, *p* < 0.01) and to the “physical activity” (r = − 0.219, *p* < 0.01), while questionnaire results reflecting medication adherence correlated with the “glucose management” (r = − 0.291, *p* < 0.01), “health-care use” subscale (r = 0.236, *p* < 0.01) and the “sum scale” (r = 0.281, *p* < 0.01). A significant difference (F = 6.225, *p* = 0.002) was found between the DSMQ mean scores of the three groups, defined by good, medium, and poor glycemic control levels.

**Conclusions:**

The Hungarian version of the DSMQ was considered a valid tool for the measurement of diabetes self-management. With its help, problematic areas of self-management could be uncovered, and interventions can be improved.

## Background

Diabetes poses an ever growing global health problem. According to estimates, there were between 340 and 536 million people living with diabetes globally in 2015, a figure predicted to rise to between 521 and 829 million by 2040 [1]. The prevalence of diabetes is growing from year to year, while HbA1c target values reflecting a controlled illness state are reached by only a third of the patients [2]. This contributes to morbidity and mortality [3], resulting in further health care, financial, and social problems, mounting to a global burden. In Hungary, based on national representative surveys, the age-standardized prevalence of self-reported type 2 diabetes increased by 89% (from 6.2 to 11.7%) between 2002 and 2012, mostly affecting working-age people [4].

Although reaching and maintaining an optimal blood glucose level depends on several factors, patient self-management in diabetes care is essential. The monitoring and strengthening of diabetes self-management, therefore, has become an aspect of utmost importance. More than 20 questionnaires have been developed in the past decade to measure self-management levels [5]. Goals of administering these questionnaires are twofold: first, they are designed to help doctors evaluate patient adherence as an adjunct to indicative clinical parameters such as HbA1c values, and second, to uncover misunderstandings about the recommended treatments [6]. However, at the time of writing, no questionnaire suitable for the assessment of diabetes self-management was available in Hungary.

As we could not find a reliable diabetes self-management questionnaire for use in Hungary, our goal was the adaptation and validation of one of the internationally available self-administered questionnaires. Based on Lu et al.’s review (2016), some questionnaires examine only one element, for instance medication adherence, while others cover several aspects of diabetes self-management. Moreover, they have not always undergone thorough clinical testing, and in addition, their psychometric profile is often unsatisfactory [5].

Out of the available questionnaires, we chose the diabetes self-management questionnaire (DSMQ) [7], developed by Schmitt et al. (2013), to adapt and validate, for three main reasons:

1. It takes into account the most important aspects of self-management: glucose management, dietary control, physical activity, and health-care use [7]
2. Available psychometric data indicate that it is a statistically reliable instrument, well-related to (HbA1c), the objective clinical indicator of diabetes control [7–9]. A study even found a detectable correlation between the DSMQ and microvascular complications, the most common consequence of poorly controlled diabetes [10].
3. It is user-friendly, short (16 items), and comprises clear and focused questions.

As the DSMQ is a relatively new instrument, few cross-cultural adaptation studies have been published yet. These studies have confirmed its reliability and clinical usefulness; however, the original factor structure either has not been confirmed [9] or has not been tested [11]. Based on the content of their items, the various subscales grasp clearly different phenomena (e.g., glucose measurement and keeping medical appointments belong to different semantical fields). Therefore, one would expect that the original four-factor model can be easily verified regardless of language. The fact that this has not been the case in previous studies has raised the possibility that the questionnaire should be considered an index, rather than a scale, in which case the factor structure depends to a great extent on the composition of the sample [12,13], which varies from study to study.

**Fig. 1:**
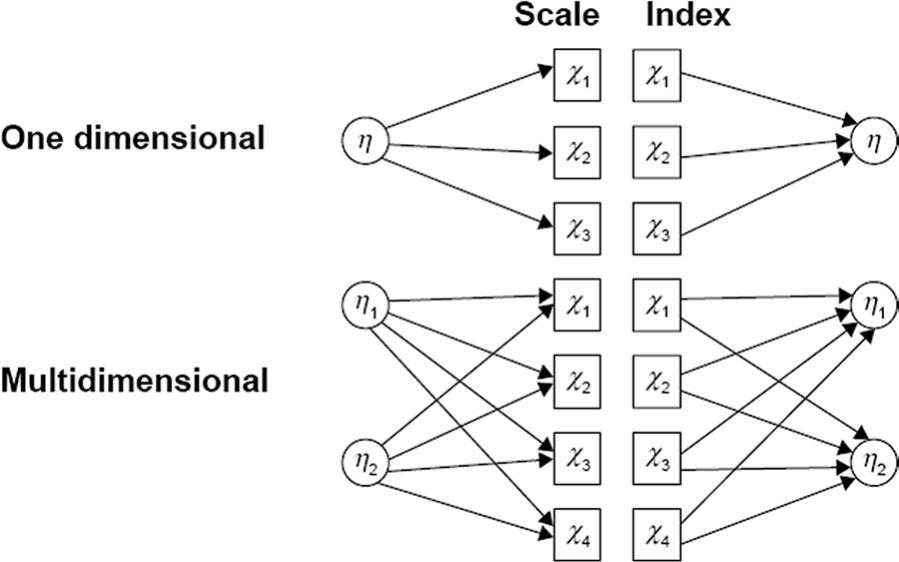
Differences in the relationship between indicators and indices vs. constructs and scales [14] (page 2084)

There is an essential conceptual difference between scales and indices. Traditional psychological instruments, such as personality inventories or depression scales, are composed of items that are intended to be manifestations of an underlying hypothetical construct [12–14]. Items of scales of this kind are called “effect indicators” [11] or “reflective indicators” [12]. In other measurement tools, typically health-related questionnaires (e.g., for assessing daily life, activity, symptoms or side effects, etc.), it is the items or subscales themselves that define the construct. The latter types of questionnaires are called indices (and not scales), and the indicators are referred to as “causal” [12,13] or “formative” [13]. In the case of effect indicators the “latent variable causes the observed variable,” while formative indicators are “assumed to cause a latent variable” [15, p. 269] (see Fig. 1).

It follows from the initial concept that inter-item correlation is essential in a conventional psychometric scale, because its items are all determined by the same construct; therefore, they should correlate with each other. In the case of indices, however, a high correlation between formative indicators would mean that they are redundant and capture very similar information [13]. Because correlation between formative items is undesirable, here it is inappropriate to use statistics based on an assumption of homogeneity of items, such as Cronbach’s alpha, principal component analysis, factor analysis, and analyses with models from item response theory [12–14]. A routine use of these psychometric methods may lead to the omission of otherwise suitable items and to a misjudgment that the index is unreliable. Instead, indices should be evaluated on the basis of how visibly/transparently (face validity) and fully they grasp the phenomenon intended to be captured, or how strongly they correlate with external criteria pointing at the same phenomenon. Furthermore, correlations among these items and the factor structure as a whole are much more dependent on the sample than is the case with scales [12,13].

As the DSMQ, like a typical health-related index, collects the most important aspects of self-management, we considered its items and subscales to be formative.

Taking the above into account and following the international consensus on adequate measurement properties for health-related patient-reported outcomes [16,17], the aim of the study was to validate the Hungarian version of the DSMQ based on (1) content validity (face validity), which is crucial for formal indicators [18], and on (2) construct validity.

## Methods

### Participants and setting

Our retrospective, cross-sectional questionnaire survey was conducted between January 1, 2017 and January 5, 2018 in a university clinic and a general hospital with the participation of consecutively recruited type 2 diabetes mellitus (T2DM) outpatients and inpatients. Ethical approval for this study was obtained from the relevant university research ethics committees. All participants provided informed consent. They were not remunerated for participation, and taking part in the study was completely voluntary.

Inclusion criteria were knowledge of the Hungarian language, being at least 18 years old, a T2DM diagnosis for at least six months, and some kind of diabetes therapy (e.g., diet, exercise) recommended by the diabetologist. Drug treatment was not an indispensable condition for participation. Exclusion criteria were inability to complete the questionnaire (e.g., because of severe cognitive impairment [dementia] or impairment of vision or reading); or any comorbid severe chronic illness (e.g., cancer, heart failure).

DSMQ translation and scoring criteria.

The diabetes self-management questionnaire (DSMQ) [7] consists of 16 statements, with four answer options each (3 = “Applies to me very much”/0 = “Does not apply to me”). Besides the sum score, scores are calculated on four subscales, covering the most important aspects of self-management: glucose management (GM, items 1, 4, 6, 10, 12), dietary control (DC, items 2, 5, 9, 13), physical activity (PA, items 8, 11, 15) and health-care use (HU, items 3, 7, 14) [7]. Item 16 is to be included in the “Sum Scale” only. The scoring of the DSMQ involves summing of all the answer scores after reversing the scores of nine negatively keyed statements. The scale scores are then transformed into a scale ranging from 0 to 10, where a score of 10 indicates the most effective self-care behavior. However, in case of missing values for more than half of the items of a scale, a scale score should not be computed.

The questionnaire was translated and linguistically validated in accordance with international guidelines [19], and it was developed in a multi-step process of forward translation, reconciliation, back translation, cognitive debriefing, and proofreading.

*Forward translation* two native Hungarian translators also proficient in English developed a forward translation independently, after which a reconciled version was agreed upon by the two translators.

*Back translation* of the reconciled version from Hungarian to English was performed by a third translator.

*Content validity* an expert panel of two diabetologists and one psychologist reviewed the translations and formulated the corrected Hungarian version of the instrument. Finally, the panel judged the face and content validity of the questionnaire.

*Cognitive debriefing* with 20 patients living with T2DM (who were not included in the study) was performed to test the interpretation of the translation. The patients’ comments were discussed by the researchers, and a summary of the changes resulting from the patient interviews was then reflected in the final Hungarian version. A significant proportion of patients indicated that, in item 2, they found the word “optimal” strange. Therefore, following their suggestion, the researchers agreed on changing the word-by-word Hungarian translation (“optimális”) into “required” (“kívánatos”). Apart from that, based on patient feedback, we changed one more expression in item 6: instead of “I record my blood sugar levels regularly” we used “I keep a regular blood sugar diary,” which is close to the original meaning but sounds more familiar in Hungarian (“rendszeresen vércukornaplót vezetek”). All other items in the Hungarian version were easily understood by the pilot group, in keeping with the simplicity and comprehensibility of the original questionnaire.

Finally, the document was proofread to check for spelling, grammar, layout, and formatting.

### Demographics and other psychological scales

The complete questionnaire package included basic demographic questions, such as age, education, and employment status. We also asked about the time since diabetes diagnosis and the patient’s knowledge of what kind of diabetic therapy had been prescribed for them, such as diet, medication, insulin, and exercise (several options could be marked). Additionally, the Morisky Medication Adherence Scale-8 (MMAS-8) was also administered, as one of the instruments widely used among diabetic patients to assess the medication adherence part of their self-management. The cross-cultural translation process into Hungarian of the MMAS-8 was previously completed (with the author’s permission), and the psychometric examination of the questionnaire is in progress.

### Clinical data

The most recent value of glycated hemoglobin (HbA1c) for the last six months was registered. As HbA1c tests are typically performed in the local lab, values come from many different labs. Body mass index (BMI = kg/m^2^) was calculated on the basis of patient-provided data.

### Statistical analyses

In order to obtain the statistical power for investigating validation and reliability, we used G*Power, a program for sample size estimation (https://www.gpower.hhu.de/), to define the appropriate sample size. According to previous results, the expected correlation coefficient could be 0.3 [7], with an α value of 0.05, a statistical power of 0.8, and two-sided testing of the sample for 82 people. To compare groups using ANOVA, 159 patients would be needed, according to G*Power software. Thus, 150–160 people should be included in the study.

The statistical analyses were performed using IBM SPSS 22.0 [20].

Demographics: To describe the demographic characteristics of the sample, we calculated frequencies and percentages for discrete variables, as well as means and deviations for continuous variables. To compare inpatients with outpatients, chi-square tests were used for discrete and independent samples t-tests for continuous variables.

*Construct validity* was evaluated via Pearson correlation of the DSMQ scale scores with (1) HbA1c values, (2) BMI values, and (3) MMAS-8 scores. A *p *value of < 0.05 (2-tailed test) was considered as a criterion of statistical significance for all analyses. Correlations were interpreted using the following criteria: 0–0.25 = weak, 0.25–0.5 = fair, 0.5–0.75 = moderate to good, and > 0.75 = very good to excellent correlation [21].

*Known-groups validity* is a method used to confirm construct validity. Known-groups validity is demonstrated when a test or questionnaire can discriminate between groups known to differ on the variable of interest [22]. We determined three categories based on the patients’ glycemic control (compared to the desirable HbA1c target value): good glycemic control (HbA1c values ≤ 7.5%), medium glycemic control (HbA1c values between 7.6% and 8.9%), and poor glycemic control (HbA1c values ≥ 9%). Then we used one-way ANOVA to compare the sum scale of the questionnaire for each glycemic control group, and each subscale (glucose management, dietary control, physical activity, and health-care use). Bonferroni post hoc analysis was applied to determine the statistical significance of differences between groups.

According to the original questionnaire scoring instructions, an item was not used if “not required as a part of my treatment” had been marked. In this case, the scale score computation was adapted by reducing the theoretical maximum score by three points [7].

The same method was used when the answer to the item was missing, that is, missing data were not replaced or imputed. In cases where more than half of the items of a scale were missing, the scale score was not computed (regardless of the reason for the absence). As suggested by the COSMIN checklist [16], we examined the missing data rate.

## Results

**Fig. 2:**
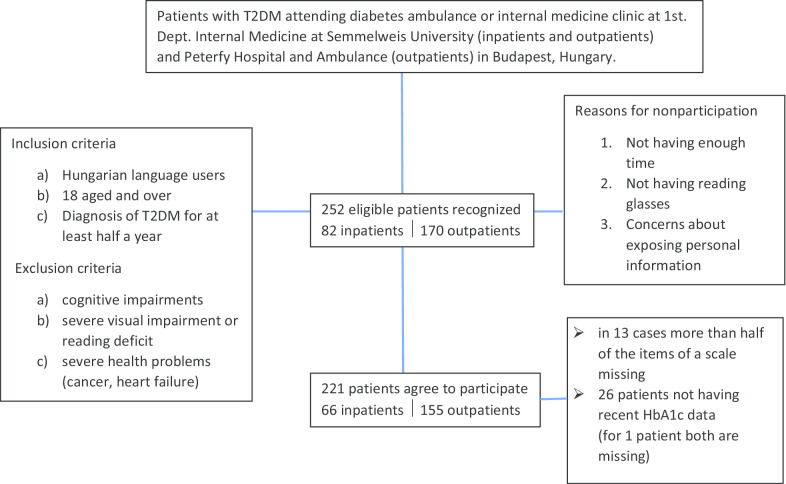
Sample structure for the Hungarian version of the Diabetes Self-Management Questionnaire (DSMQ) validation

Out of the 252 participants eligible for this study, 221 patients agreed to complete the questionnaire (willingness to participate was 88%). Thus, 221 patients’ data (66 inpatients and 155 outpatients) were statistically analyzed (see Fig. 2).

The most common reasons for refusal to participate were lack of time, lack of reading glasses, and concerns about sharing personal information. For 13 people, more than 50% of the data in the questionnaire were missing. According to the scoring rules for the original version of the questionnaire [7], these respondents were excluded. Twenty-six subjects had to be excluded because they failed to provide their HbA1c results to their diabetologist. One patient provided neither sufficient questionnaire data nor HbA1c results.

### Clinical and demographic data

Our sample consisted of slightly more men than women (129/92), and 68% of the sample were aged over 60, inactive, middle-income, with BMIs falling into the obese category (BMI > 30) (see Table 1).

**Table 1:** Characteristics of the study sample (N = 221)

Parameter	Patients (N = 221)	Inpatients (N = 66)	Outpatients (N = 155)
	n (%) or mean ± SD		
Gender			
Male	129 (59)	42 (64)	87 (56)
Female	92 (41)	24 (36)	68 (44)
Age (years)	63 ± 11.89	65.3 ± 10.17	62 ± 12.44
18–45	14 (6)	3 (4)	11 (7)
46–60	56 (26)	17 (26)	39 (25)
> 60	151 (68)	46 (70)	105 (68)
BMI	32.6 ± 6.44	32.4 ± 5.8	32.7 ± 6.7
Education			
Primary	20 (9)	11 (17)	9 (6)
Secondary	126 (57)	39 (59)	87 (56)
University	75 (34)	16 (24)	59 (38)
Employment status			
Active (employed)	81 (37)	18 (27)	63 (41)
Inactive (unemployed/retired)	140 (63)	48 (73)	92 (59)
Time since diagnosis			
≤ 1 year	15 (6)	3 (5)	12 (8)
1–3 years	11 (5)	4 (6)	7 (5)
3–5 years	30 (14)	6 (9)	24 (15)
> 5 year	165 (75)	53 (80)	112 (72)
Diabetes therapy			
Medication no./day	2.5 ± 3.1	2.7 ± 4.5	2.4 ± 2.3
Units of insulin/day	1.66 ± 1.8	1.65 ± 1.8	1.67 ± 1.8
Diet needed	177 (80)	45 (68)	132 (85)
Physical activity need	149 (67)	34 (51)	115(74)
HbA1c value*	7.2 ± 1.39	7.74 ± 1.85	7.02 ± 1.11
MMAS-8	6.86 ± 1.26	6.6 ± 1.36	6.97 ± 1.2
DSMQ “Sum scale”*	7.65 ± 1.23	7.13 ± 1.28	7.85 ± 1.15
Subscale “Glucose management”*	7.9 ± 1.74	7.37 ± 1.76	8.2 ± 1.69
Subscale “Dietary control”	6.97 ± 1.65	6.96 ± 1.84	6.9 ± 1.55
Subscale “Physical activity”*	6.47 ± 2.59	5.9 ± 2.48	7.06 ± 2.58
Subscale “Health-care use”*	8.84 ± 1.68	7.8 ± 1.93	9.25 ± 1.39

^*^Significant differences between inpatient and outpatient groups (Significant differences *p* < 0.05; Nonsignificant differences *p* ≥ 0.05)

Two-thirds of study participants (75%) had been diagnosed with T2DM for more than five years. No significant difference was detected between inpatients and outpatients concerning the amounts of oral antidiabetics and the number of daily insulin units taken. A comparable rate of inpatients (68%) and outpatients (85%) considered diet as being a part of their treatment. Outpatients considered exercise as a necessary requirement more often than inpatients, i.e. 74% versus 51%; but the difference was not significant. However, the mean of the HbA1c values was significantly different between the inpatient and outpatient groups: the HbA1c value of the outpatient group was closer to the target value (inpatient group: 7.74 ± 1.85, outpatient group: 7.02 ± 1.11), which in this case is not at all surprising, since many of the inpatients were hospitalized precisely for the purpose of having their carbohydrate metabolism fixed.

Diabetes self-management scores differed between inpatients and outpatients on the sum scale and on the glucose management, physical activity, and health-care use subscales, but not on the dietary control subscale. Ranges and percentages of respondents scoring maximum points are the following: sum scale (range = 4.58–10, maximum-point percentage 9%), glucose management (range = 2.2–10, maximum-point percentage 21.3%) dietary control (range = 2.5–10, maximum-point percentage 6.3%), physical activity (range = 0–10, maximum-point percentage 19.9%), and health-care use subscale (range = 2.2–10, maximum-point percentage 53.4%).

Missing data occurred in 7.4% of the cases (16.3 [mean per item]/221), and in 13 cases out of 221 (5.9%), scale scores were not computed because more than half of the items were missing.

### Construct validity

The subscales of the questionnaire and the sum scale total score showed little or fair correlations with HbA1c, except for the physical activity subscale. The strongest inverse correlation was found between the DSMQ sum scale and the HbA1c scores (r = − 0.253, *p* < 0.01). Glucose management (r = − 0.156, *p* < 0.05), dietary control (r = − 0.230, *p* < 0.01), and health-care use (r = − 0.227, *p* < 0.01) subscales were significantly correlated with HbA1c values (see Table 2). BMI was most strongly related to the sum scale (r = − 0.214, *p* < 0.01) and showed a significant inverse relationship with the physical activity subscale (r = − 0.219, *p* < 0.01). In addition, BMI had a low but significant correlation with glucose management (r = − 0.148, *p* < 0.05) (see Table 2). The MMAS-8 showed a fair correlation with glucose management (r = 0.291, *p* < 0.01) and with the sum scale (r = 0.281, *p* < 0.01); and a weak correlation with the health-care use (r = 0.236, *p* < 0.01) and the dietary control (r = 0.174, *p* < 0.05) subscales (see Table 2).

**Table 2:** Correlations of DSMQ scales and HbA1c, BMI, MMAS-8

	DSMQ scales				
	SS	GM	DC	PA	HU
HbA1c	− 0.253**	− 0.156*	− 0.230**	ns	− 0.227**
BMI	− 0.214**	− 0.148*	ns	− 0.219**	ns
MMAS-8	0.281**	0.291**	0.174*	ns	0.236**

HbA1c, glycated hemoglobin; BMI, body mass index; MMAS-8, Morisky Medical Adherence Scale; SS, sum scale; GM, glucose management; DC, dietary control; PA, physical activity; HU, health-care use

^**^Correlation is significant at the 0.01 level (2-tailed)

^*^Correlation is significant at the 0.05 level (2-tailed)

### Known-groups validity analysis

There was a difference between the DSMQ sum scale means of patients with well-controlled, medium-controlled, and poorly-controlled glycemic levels (F = 6.225, *p* = 0.002). Significant differences were found between the medium- and poorly controlled groups (mean difference (MD) = 0.89, SD = 0.33, *p* = 0.023), and also the well- and poorly controlled groups (MD = 1.04, SD = 0.29, *p* = 0.002). There was no significant difference between the well- and medium-controlled groups (see Table 3).

**Table 3:** The relationship between DSMQ scales and glycemic control groups

	Glycemic groups			Sign. (*p* =)
	Well-controlled (HbA1c ≤ 7.5%)	Medium-controlled (7.6% < HbA1c > 8.9%)	Poorly-controlled HbA1c ≥ 9%	
N (%)	122 (67)	42 (23)	19 (10)	
DSMQ “Sum scale” Mean (± SD)	7.79 (± 1.17)	7.64 (± 1.18)	6.75 (± 1.8)	** (0.002)
“Glucose management” Mean (± SD)	8 (± 1.68)	7.93 (± 1.76)	7.19 (± 2.01)	ns
“Dietary control” Mean (± SD)	7.18 (± 1.67)	6.76 (± 1.22)	6.13 (± 1.94)	* (0.021)
“Physical activity” Mean (± SD)	6.82 (± 2.44)	6.87 (± 2.69)	5.9 (± 3.33)	ns
“Health-care use” Mean (± SD)	9.04 (± 1.51)	8.75 (± 1.9)	7.77 (± 1.97)	** (0.008)

Significance **p < 0.01; *p < 0.05, *ns* not significant

There was a significant difference in two of the subscales, dietary control (F = 3.944, *p* = 0.021) and health-care use (F = 4.91, *p* = 0.008), among the three glycemic control groups. The poorly controlled and the well-controlled groups were significantly different from each other in terms of both dietary control (MD = 1.05, SD = 0.39, *p* = 0.028) and health-care use (MD = 1.26, SD = 0.41, *p* = 0.007). There were no significant differences in the other subgroups for any of these subscales. Neither the glucose management nor the physical activity subscales differed significantly along HbA1c categories (see Table 3).

## Discussion

The aim of this study was the validation of the diabetes self-management questionnaire (DSMQ, [7]) in a Hungarian translation and within a Hungarian clinical population. Regarding the nature of the questionnaire, we adopted an approach different from the original, because in our view the formative conception is a better fit.

Our sample consisted of average in- and outpatients with T2DM. Most of them had a BMI above the upper limit of the normal range, were aged over 60, and were economically inactive, which is typical for this patient population. The inpatient group had higher HbA1c means compared to the outpatient group, which was not surprising given that inadequate blood glucose management can be the cause of hospitalization. Except for the dietary control subscale, all DSMQ subscales and also the sum scale proved to be significantly higher in the outpatient group. This, again, may logically lead to the conclusion that the self-management of the outpatient group was better, necessitating no hospitalization for carbohydrate metabolism regulation. Thus, the answers elicited by the questionnaire seem to adequately reflect the construct intended to be measured.

The present study proves a satisfactory construct validity of the DSMQ with an objective clinical parameter, the HbA1c value. We found a significant but weak inverse correlation between the DSMQ sum score and the HbA1c values (r = − 0.253) that is very similar to the original questionnaire’s correlation with HbA1c (− 0.23 ± 0.09) [7]. However, it must be mentioned that it would be unrealistic to expect a strong correlation, as HbA1c is not a one-to-one reflection but a fair proxy of self-management. Glucose level is not the only biological parameter affecting HbA1c concentration; it might be influenced by anemia, acute inflammation, age, and so on [23]. Also, one has to take into consideration the phenomenon called “therapeutic inertia,” as diabetologists have a tendency to delay the intensification of the therapy when HbA1c levels remain higher than desirable [24].

The relationships between BMI values and the DSMQ also confirmed the convergent validity of the questionnaire. As presumed, BMI was inversely correlated with the sum score (r = − 0.214), physical activity (r = − 0.219), and glucose management (r = − 0.148) subscales, although the correlations were low. Similarly, the results of the MMAS-8 showed a positive correlation (r = 0.291) with the glucose management subscale, which partly tests medication adherence. These findings verify the construct validity of the Hungarian DSMQ instrument.

Based on the result of the known-groups validity test, we can say that the questionnaire only roughly differentiated between glucose control subgroups. In the population studied, there were significant differences in DSMQ sum scores between the well- (HbA1c values ≤ 7.5%) and poorly controlled (HbA1c values ≥ 9%) groups, and between the medium- (7.6% < HbA1c > 8.9%) and poorly controlled groups, but no significant differences between the good and medium glycemic control groups. Similar differences were found according to the dietary control and the health-care use subscales, but no difference was found between groups on the glucose management (GM) and physical activity (PA) subscales, which is similar to the original validation of the questionnaire where there were no significant differences on any subscale along HbA1c categories [7]. Thus, questionnaire scores were clearly related to the HbA1c values but could differentiate only between patient groups with acceptable versus extremely high HbA1c values.

This study has some potential limitations. We observed a ceiling effect in the case of the health-care use subscale but also to some extent for the GM and PA subscales. While every fifth person scored maximum points in GM and PA, in health-care use this was true for as much as half of the respondents.

The ceiling effect can be related to the fact that the questionnaires were completed immediately before the medical visit, which may distort the responses, especially those referring to regular medical appointments. Further, due to the design of the study, we do not have data from people not keeping appointments or not going to laboratory tests. All of this might significantly affect the quality of the data and might partly explain the finding that there was only a weak relationship between the subscales and the HbA1c categories.

In addition, we used HbA1c values registered over a six-month period, while behavioral changes can occur within a shorter period of time. The optimal method would have been to measure HbA1c, with participants simultaneously completing the questionnaire. Due to financial reasons, this procedure was not possible. Moreover, HbA1c results came from many different laboratories, and therefore a slight lab measurement bias cannot be ruled out. This can result in correlations with HbA1c falling short of those measured in previous studies. Another potential source of bias can be that BMI was calculated on the basis of self-reported weight and height data. There were advantages to examining both outpatient and inpatient groups with potentially different characteristics and diabetes-related habits, but this may also have caused some inhomogeneity in the data.

## Conclusion

To sum up, these results indicate that the Hungarian version of the self-administered DSMQ is suitable for clinical use, as it can help physicians to make more nuanced adherence judgments, thus contributing to the effectiveness of diabetes care. Although it can help to detect severe adherence problems, it has limited predictive validity regarding HbA1c values. Nevertheless, the availability of the Hungarian version of the DSMQ may inspire further scientific studies in the field.

## Supplementary information

**Additional file 1.** Result of back translation (from Hungarian to English) of the Diabetes Self-Management Questionnaire (DSMQ).

## Data Availability

The data set used and/or analyzed during the current study are available from the corresponding author on reasonable request.

## Abbreviations

BMI: body mass index
DC: dietary control
DSMQ: diabetes self-management questionnaire
GM: glucose management
HbA1c: glycated hemoglobin
MMAS-8: Morisky Medication Adherence Scale
PA: physical activity
SS: sum scale
T2DM: type 2 diabetes mellitus
