# Supplementary material for: The validity of the diabetes self-management questionnaire (DSMQ) in Hungarian patients with type 2 diabetes
Source: Health Qual Life Outcomes. 2020 Oct 19;18:344. doi: 10.1186/s12955-020-01595-7 (PMC7574306; doi:10.1186/s12955-020-01595-7)
Supplement: Supplementary file 1 — Additional file 1. Result of back translation (from Hungarian to English) of the Diabetes Self-Management Questionnaire (DSMQ). [file 12955_2020_1595_MOESM1_ESM.docx]

**Additional file 1: Result of back translation (from Hungarian to English) of the Diabetes Self-Management Questionnaire (DSMQ)**

| **Below you will find a set of statements regarding the self-management of your diabetes. Based on the past 8 weeks how true are the statements for you?** | (3) | (2) | (1) | (0) |
| --- | --- | --- | --- | --- |
| 1. I check my blood sugar level regularly and with care.   *□^^[[1]](#footnote-1)^^ Blood sugar measurement isn’t part of my treatment* | 3 | 2 | 1 | 0 |
| 1. I achieve optimal blood sugar levels with my regular diet | 3 | 2 | 1 | 0 |
| 1. I keep my check-up appointments with my doctor | 3 | 2 | 1 | 0 |
| 1. I take my diabetes medication(tablets/insulin) as ordered by my doctor.   □ I don’t take medication for my diabetes | 3 | 2 | 1 | 0 |
| 1. On occasion I eat lots of sweets or other foods containing high carbohydrates. | 3 | 2 | 1 | 0 |
| 1. I regularly write down my blood sugar levels (save them in the measuring device).   □ *Blood sugar measurement isn’t part of my treatment.* | 3 | 2 | 1 | 0 |
| 1. I tend to avoid diabetes doctors’ appointments. | 3 | 2 | 1 | 0 |
| 1. I exercise regularly in order to achieve optimal blood sugar levels. | 3 | 2 | 1 | 0 |
| 1. I strictly follow the diet advised by my doctor or dietician. | 3 | 2 | 1 | 0 |
| 1. I don’t take my blood sugar regularly enough to achieve good blood sugar control.   □ *Blood sugar measurement isn’t part of my treatment.* | 3 | 2 | 1 | 0 |
| 1. I avoid physical activity even though it could help in my diabetes treatment. | 3 | 2 | 1 | 0 |
| 1. I tend to forget to take my diabetes medications.   □ I don’t take medication for my diabetes*.* | 3 | 2 | 1 | 0 |
| 1. Sometimes I binge eat (not because of hypoglycaemia). | 3 | 2 | 1 | 0 |
| 1. I should visit my doctor more often for my diabetes treatment. | 3 | 2 | 1 | 0 |
| 1. I tend to skip planned physical exercises. | 3 | 2 | 1 | 0 |
| 1. I don’t take proper care of my diabetes treatment. | 3 | 2 | 1 | 0 |

**Additional file 2: DSMQ (Diabetes Self-Management Questionnaire) - Hungarian**

**
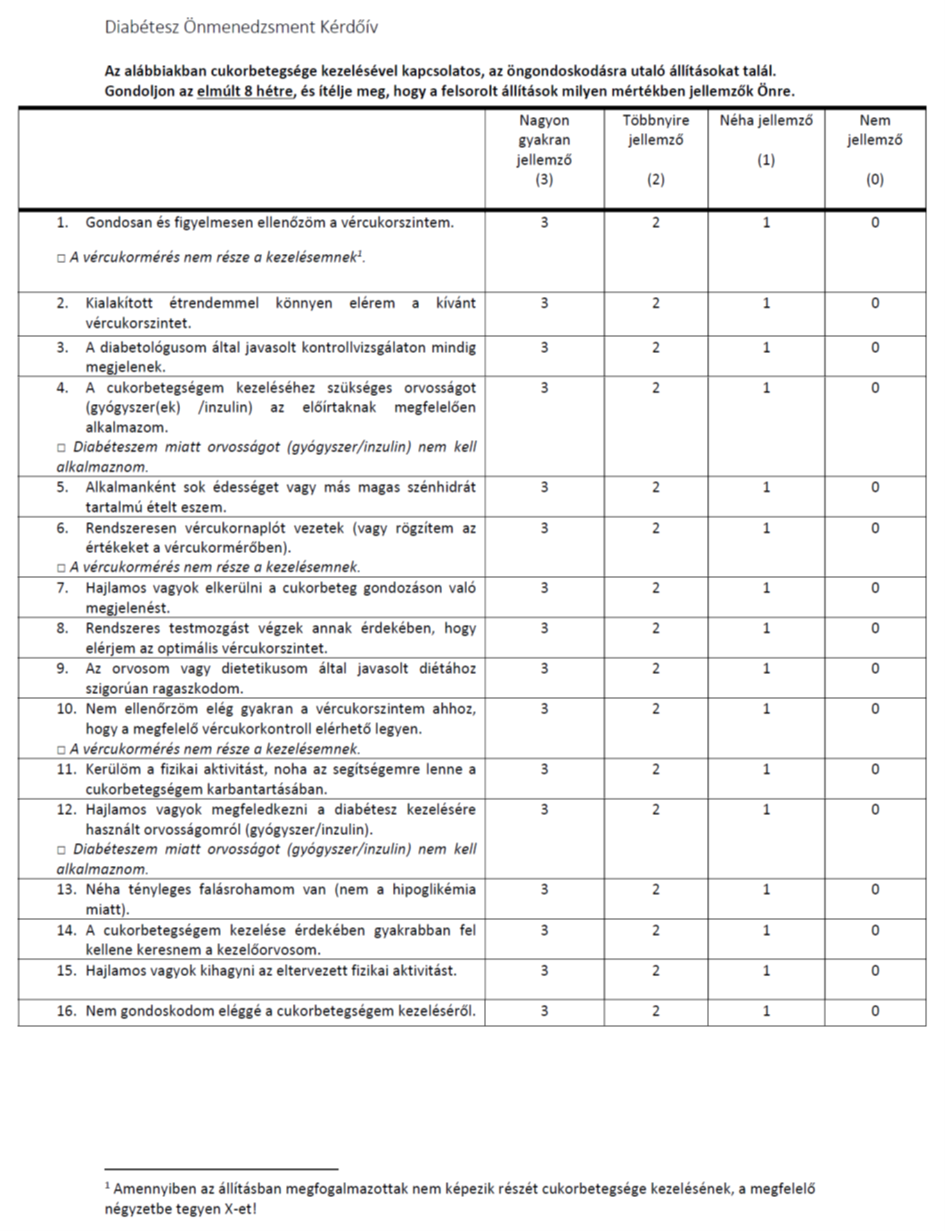
**

1. Amennyiben az állításban megfogalmazottak nem képezik részét cukorbetegsége kezelésének, a megfelelő négyzetbe tegyen X-et! [↑](#footnote-ref-1)
